# Investing in AI for social good: an analysis of European national strategies

**DOI:** 10.1007/s00146-022-01445-8

**Published:** 2022-05-05

**Authors:** Francesca Foffano, Teresa Scantamburlo, Atia Cortés

**Affiliations:** 1 University of York, York, UK; 2 European Centre for Living Technology, Venice, Italy; 3 Ca’ Foscari University of Venice, Venice, Italy; 4 Barcelona Supercomputing Center, Barcelona, Spain

**Keywords:** AI policy, AI for social good, European National Strategy, AI investments, Trustworthy AI

## Abstract

Artificial Intelligence (AI) has become a driving force in modern research, industry and public administration and the European Union (EU) is embracing this technology with a view to creating societal, as well as economic, value. This effort has been shared by EU Member States which were all encouraged to develop their own national AI strategies outlining policies and investment levels. This study focuses on how EU Member States are approaching the promise to develop and use AI for the good of society through the lens of their national AI strategies. In particular, we aim to investigate how European countries are investing in AI and to what extent the stated plans contribute to the good of people and society as a whole. Our contribution consists of three parts: (i) a conceptualization of AI for social good highlighting the role of AI policy, in particular, the one put forward by the European Commission (EC); (ii) a qualitative analysis of 15 European national strategies mapping investment plans and suggesting their relation to the social good (iii) a reflection on the current status of investments in socially good AI and possible steps to move forward. Our study suggests that while European national strategies incorporate money allocations in the sphere of AI for social good (e.g. education), there is a broader variety of underestimated actions (e.g. multidisciplinary approach in STEM curricula and dialogue among stakeholders) that can boost the European commitment to sustainable and responsible AI innovation.

## Introduction

The integration of Artificial Intelligence (AI) into social life has raised concerns worldwide and efforts to limit the negative impact associated with the use of such systems have multiplied in recent years. Research communities, non-profit organisations and governmental agencies have stressed the point that AI is not “simply” a technological artifact. AI is a socio-technical system made of an imprecise number of heterogeneous components ranging from pieces of code, high-performing CPUs to human assumptions and social habits.

Although the complex interdependencies underlying an AI system makes it difficult to fully predict and control its behavior, there is a large consensus on steering AI development towards the good of people and the environment. In this regard, a truly global effort is the movement of AI for Social Good, a flurry of AI-related activities aimed at delivering positive social impact (Tomašev et al. 2020; Floridi et al. 2020). These include AI projects addressing specific social problems, such as hate speech or climate change, as well as guidelines and frameworks offering guidance on the implementation of safe and ethical AI.

However, what counts as AI for social good is still debated both theoretically and practically (Floridi et al. 2020). The simple availability of AI systems with socially good outcomes might not guarantee that they are being used for the common good as ethics guidelines would recommend (HLEG AI 2019a). For example, certain applications can originate from a genuine aspiration to solve a social problem (e.g. student dropout or prison overcrowding) and then generate unexpected harms or inconveniences as they go live (e.g. outcomes benefiting a small portion of the population or users’ over reliance). Ethics principles, moreover, will remain a sterile exercise if we do not put them into practice. Good AI policies are essential to support a more practical approach to ethical principles whose impact depends on how they integrate into larger governance ecosystems including relevant policies, laws, regulations and existing practices (Fjeld et al. 2020). In addition, AI policies can operate at different scales and impact on a variety of stakeholders thereby promoting a more inclusive and responsible innovation approach across all ramifications of our society. For example, education and vocational training constitute a key area of intervention for a successful undertaking of AI for social good. Organisations and governments planning measures in this sphere give greater support to future and present workers in dealing with the challenges and opportunities brought by AI.

In this paper, we focus on European AI policies and how these relate to AI for social good. We want to explore how Member States introduce AI for social good in their national strategies. In particular, we focus on their investment plans and how these commit to the human-centric approach proposed by the European Commission (EC) and embedded into European Guidelines for Trustworthy AI. Our choice is motivated by the assumption that information about concrete investments can be a meaningful indicator of countries’ commitment towards AI for social good and the European human-centric vision. Therefore the identification of concrete measures presented in the strategies will help to envision how European countries are planning to fulfill the EC projections on AI. Our guiding questions are: What do Member States plan to do for a responsible development of AI? Do they translate the human-centric vision into targeted measures? What plans do they have to make AI development more democratic and open to society? In other words, is AI made in Europe truly fostering social good? We will try to answer these questions by analysing the investment plans stated in the European national strategies and providing a critical reflection on the definition of AI for social good using the EU AI Policy as a reference. The aim is to identify general trends and types of investments that can foster the development of a beneficial and sustainable AI. While our analysis suggests that Member States are trying to make concrete steps towards socially good AI, there is room for reflection on the maturity of their efforts.

The article is structured as follows. Section 2 introduces the idea of AI for social good, highlighting the role played by AI policies, and provides an overview of the European AI strategy and related key initiatives. In Sect. 3 we dig into the European Member States’ strategies and analyse the investment plans stated in such documents. Section 4 describes the results of our analysis providing an overview of the general trends. In Sect. 5 we comment on our findings based on the policy directions taken by the EU on AI for social good. Finally, we provide some concluding remarks summarising the work done and highlighting some open issues.

## Moving towards a good AI society

The field of AI for social good spans a variety of stakeholders and actions such as public events,^1^ scientific publications,^2^ movements and organisations’ special programmes.^3^

In the following sections, we will explore the notion of AI for social good at the semantic level by outlining the meanings that usually relate to it. In particular, we will focus on the policy element, which can guide and complement other components (i.e. AI applications with a socially good outcome and ethical principles), and provide an overview of the European strategy towards AI. The latter is, in fact, an example of a policy approach that aims to be “human-centric” and for the well-being of society, and, thus, it is a source of stimuli for discussing how strategic choices can influence the implementation of AI for social good.

### Framing AI for social good

What do we mean by “AI for Social Good”? To answer this question we identify three possible and complementary meanings.

1. AI for Social Good as applications

A first, intuitive meaning is to achieve a positive impact by applying AI to societal and environmental challenges such as natural disaster management, poverty reduction and climate change. For example, Climate Change AI is an organisation of volunteers using machine learning to help reduce gas emissions. At Stanford University a group of researchers combined publicly available satellite imagery with deep learning to predict the level of poverty across African villages and support organizations to deliver services to those most in need (Yeh et al. 2020). Usually, a shared objective of these and similar projects is to advance social good as defined by the UN Sustainable Development Goals (SDGs) (The Future Society 2020). However, note that applying AI to a specific social or environmental challenge alone is not a guarantee for the achievement of social good. For instance, a study on the impact of AI on the achievement of the UN’s Sustainable Development Goals revealed that AI can act as an enabler on 79% of sustainable development targets but also inhibits 35% of them (Vinuesa et al. 2020).

Other challenges include AI for social good applications resulting in a failure because of poor ethical considerations (the so-called “good-AI-gone-bad”) or reflecting an accidental success which misses the opportunity to extend the positive effects of AI to other settings (Floridi et al. 2020). To address these issues, Floridi et al. (2020) propose seven ethical factors that provide both theoretical and practical guidance for the design and implementation of AI projects in agreement with well-known ethical principles (beneficence, non maleficence, justice, autonomy, and explicability).

2. AI for Social Good as ethical principles

Another way to spell out the meaning of AI for social good is to define a set of principles that can inspire the design and assessment of AI systems. Popular examples include the European Guidelines for Trustworthy AI (HLEG 2019a) and the intergovernmental principles adopted by the Organisation for Economic Co-operation and Development (OECD 2019). Similar efforts have produced an impressive literature in the last few years and extensive reviews suggest a meaningful convergence around common themes, such as accountability and non-discrimination, but also the need for more concrete approaches making such principles practically relevant (Fjeld 2020; Hagendorff 2020; Jobin et al. 2019; Zeng et al. 2018). Whittleston et al. (2019) argue that a way to move principled AI forward is to acknowledge and address the tensions that arise when applying ethical principles in practice. Many scholars developed specific tools and methodologies supporting the implementation of ethical principles in the deployment and assessment of AI systems, and recent works have provided classification, more or less articulated, of available tools (Morley et al. 2019; Scantamburlo et al 2020).

3. AI for Social Good as policies

A third important strand in the field of AI for social good is the development of AI policies which set up priorities and action plans for the development and adoption of AI in the public interest. Note that “policy” is a general term that may apply to different entities, such as firms and associations, but here we focus on public policy, i.e. those adopted by states to harness the potential of AI and protect citizens. In broad terms, an AI policy refers to a plan of actions which addresses both the opportunities and the risks brought about by AI. Usually, it includes statements about resource allocations and commitments to specific areas of concerns (Mattingly-Jordan et al. 2019). It may inspire new laws or solicit changes to current regulations in response to specific AI characteristics. In general, a policy “conveys the necessity of exploration and planning, the finality of law, and the primacy of public interest without definitely endorsing or rejecting regulatory intervention” (Calo 2017).

AI policies can play a key role in the field of AI for social good complementing and integrating the other two elements (i.e. AI applications and ethical principles). First, they can offer a roadmap for a wide range of actors (companies, non-profit organisations, research centres, public administrations, individual citizens, etc.) offering guidance and bringing cohesion to a variety of initiatives and projects. Second, AI policies translate ideals and goals into action plans providing concrete information on fundamental aspects such as funding and investments, governance mechanisms, areas of development and risks mitigation. Although policy documents may contain partial or provisional information, they can work as a mirror reflecting the values and the priorities of a government, but also the incoherences and the gaps between the aspirations and the planned actions. Nonetheless, the choice to approach AI for social good from a policy perspective does not exclude other conceptualizations, such as those described above. In fact, our idea of AI for Social Good cannot be reduced to a single dimension. AI projects that implement UN SDGs are genuine examples of AI for social good. However, we also acknowledge that setting up AI for social good policies is paramount to give an agenda soliciting and steering more concrete initiatives. In this paper, we highlight the policy dimension because it can offer new, fresh insights to the field of AI for social good.

Since 2017, 60 countries, territories and the EU have published over 700 AI policy documents,^4^ also known as National or Regional AI Strategies, to set out their vision on AI, define specific interventions and coordinate governmental or intergovernmental efforts (for an inventory of these documents see Van Roy 2020; Zhang et al. 2021). In the following section, we direct our attention towards the European AI strategy to better understand the priorities and initiatives undertaken by the European Union to steer the development of AI. This overview, moreover, will set the stage for our analysis of Member States’ strategies.

### The European strategy

In the last few years, the development and the deployment of AI have grown dramatically at a global scale. While some top players such as China and the United States are moving faster to achieve leadership in AI, focusing mostly on the industry, the vision of the European Union (EU) is to bring a balance between innovation and ethical sustainability. In particular, the ambition stated by the EC is: “to become the world-leading region for developing and deploying cutting-edge, ethical and secure AI, promoting a human-centric approach in the global context.” (European Commission 2018c).

To move towards this goal, the EC put forward specific initiatives including policy documents, ethics guidelines and, more recently, a proposal for a regulation (European Commission 2021a; b). All these efforts aim to define a unified approach across all EU Member States and ensure responsible AI innovation. The ethics guidelines for Trustworthy AI represent a milestone in the European pathway which has involved an articulated production process (Smuha 2019). A more recent effort has been the preparation of a regulatory framework, first announced in a white paper (European Commission 2020a), and now officially proposed in a EC’s communication (European Commission 2021a; b) specifying a set of rules based on AI systems’ risk level.

The aforementioned initiatives are part of a wider process which is guided by three goals defining the pillars of the European AI strategy (European Commission 2018b):

1. Boost the EU’s technological and industrial capacity and AI uptake across the economy by private and public sectors. This implies to strengthen research and development investments in AI in the EU.
2. Prepare for socio-economic changes brought by the transformation of AI in the labor market. Member States will need to prepare the Society to develop basic digital skills; re-skill or up-skill workers affected by automation, robotics and AI; and train more AI specialists, aiming for academic excellence.
3. Ensure an appropriate ethical and legal framework to promote trustworthy and accountable AI made in Europe.

Table 1 provides an overview of the main takeaways from fundamental policy documents (for an overview of the EU AI landscape see Stix 2019). Surveying the recommendations set out in these documents we identified some macro-areas of policy directions that can help us contextualise the analysis of national strategies and their investment plans (see Sect. 3):

- *Beneficial innovation* to improve public services and tackle social problems: e.g. the use of AI for policy development and sustainable challenges (such as reducing pesticides in agriculture), more efficient and targeted interventions in the public sectors (e.g. healthcare, employment, security, etc.), accessibility of AI services to the whole society including protected groups.
- *Education and life-long learning* to prepare the society for an AI future: e.g. access to AI literacy in primary and secondary schools, courses for up- or re-skilling, increasing gender balance in AI and STEM, introduction of non-technical skills in AI and STEM curricula.
- *Governance mechanisms* to steer AI towards a beneficial use aligned with human rights and the rule of law through the elaboration and adoption of ethics guidelines and legal framework, systems to continuously monitor the impact of AI on society.
- *Cooperation and dialogue among stakeholders:* e.g. exchange of best practices, creation of multi-stakeholder alliances, facilitating partnerships (e.g. private and public) and research networks also to maximize investments and facilitate technology transfer.
- *Pan-European data and technological infrastructure* including the creation of trusted data spaces, the provision of testing facilities and sandboxes, and investments in clouds and high-performance computing, among others.

**Table 1:** Takeaways of the European policy documents on AI from 2018 to 2020

Document	Takeaways
Declaration of cooperation on Artificial Intelligence*, April 2018* (EC 2018a)	Addressing the transformation of the labour market and modernising Europe’s education and training: Encouraging discussion with stakeholders Exchanging views on ethical and legal frameworks Ensuring that humans remain at the centre of the development, deployment and decision-making of AI Advancing public understanding of AI Exchanging views on the impact of AI on the labour market
Artificial Intelligence for Europe*, April 2018* (EC 2018b)	The EU strategy aims to (EU pillars): Boost the EU’s technological and industrial capacity and AI uptake across the economy by private and public sectors Prepare for socio-economic changes brought by the transformation of AI in the labour market Ensure an appropriate ethical and legal framework to promote trustworthy and accountable AI made in Europe
Coordinated Plan on Artificial Intelligence*, December 2018* (EC 2018c)	Strategic actions such as: Recommendation for all Member States to develop national AI strategies outlining investment levels and implementation measures Access to the necessary AI and ICT skills in primary and secondary schools Ethics and other non-STEM skills should be part of the talent fostering chapter of AI national and international strategies Exchange best practices on how to reinforce excellence and to retain AI talent in Europe and on the re- and upskilling of the current workforce Creation of a common European data space accessible to a broad range of users in full respect of GDPR and based on shared standards Use of AI to improve public services (e.g. detection of criminal activities) and facilitate citizen-government interaction Increasing international cooperation to promote ethics guidelines and integrate AI into development policy
Policy and Investment recommendations of AI *June 2019* (HLEG AI 2019b)	Recommendations associated with four areas where AI can bring a positive impact (civil society, private sector, public sector, and research and academia), such as: Increasing digital and AI literacy through courses (e.g. MOOCs) Encouraging the development of AI tools and applications that are specifically targeted to help vulnerable demographics Monitoring mechanisms at national and EU level to continuously analyse, measure and score the societal impact of AI Gaining access to data and infrastructure for developing welfare-enhancing AI solutions through privacy-preserving means Stimulating beneficial innovation by funding EU hackathons, competitions and industry challenge-driven research missions in AI Recommendations linked to four enablers (data and Infrastructure, Education and Skills, a Governance and Regulatory framework and Funding and Investment): Network of testing facilities and sandboxes with appropriate governance mechanisms to set legal and ethical standards Fostering the creation of trusted data spaces for specific sectors (e.g. healthcare) and the creation of AI-based services that are available for all Promoting skills related to data and AI in all academic disciplines and professional fields to increase the potential of areas where AI applications can be developed Incorporating humanities, social sciences, and gender research into AI research programmes to increase diversity and guarantee a multidisciplinary approach
White Paper on AI: a European approach to excellence and trust*, 2020* (EC 2020a)	Policy options to enable a trustworthy and secure development of AI: Mobilising resources to achieve an ‘ecosystem of excellence’ and create the right incentives to accelerate the adoption of solutions based on AI Outlining the elements of a regulatory framework for AI in Europe that will create a unique ‘ecosystem of trust’

The policy directions outlined above aim to achieve the goals set out in the European AI strategy (see the three pillars) and try to contribute to sustainable innovation. In addition, each policy area suggests measures that can foster AI for social good and contribute to build a roadmap for our quality analysis of the investment plans stated in National AI strategies.

In addition to policy documents, The EU strategy is supported by other initiatives such as collaborations, projects and funding opportunities. Some activities facilitate the monitoring of AI development along different dimensions including industrial, technological and research capacity and policy initiatives, such as the AI Watch^5^ (JRC). While others promote cooperation through industrial and research networks such as the Digital SME Alliance,^6^ Claire,^7^ Ellis,^8^ and EurAI.^9^

Among projects, the AI4EU^10^ platform fosters collaboration among AI actors, sharing expertise and research at the European level. The platform also includes the European Observatory on Society and AI, which is coordinated by the authors of this work. The goal of the Observatory is to present and reflect on the development of AI in Europe, in particular regarding the ethical, legal, social, economic and cultural aspects of AI. This paper results from the collection of EU policy documents carried out by the AI4EU Observatory team and incorporate the reflections developed and shared in this research context.

## European national strategy analysis

As we saw, the European AI strategy is premised on the idea that AI should be a “force for good in society with the ultimate aim of increasing human well-being.” (European Commission 2021a; b, p. 1). Moreover, the goals and the clusters of policy proposals identified in key EU documents (see Sect. 2.2) set out priorities and action plans that can facilitate the implementation of AI for the social good.

This paper aims to analyse how the goals and the policy directions of the European AI strategy translate in practice. In particular, we will examine what Member States plan to allocate resources in their AI national strategies. Are they promoting AI for social good?

To answer this question we analysed 15 European national policies to understand how Member States plan to contribute to the achievement of Trustworthy AI. In particular, we focus on investment plans to see whether the ethical and social aspiration emerging from the European AI policy documents translates into concrete actions and specific resource allocations.

In the following sections, we contextualise our work and illustrate similar research comparing recent ethical guidelines and frameworks. We then describe the documents studied, also from a quantitative perspective, and present the selection process as well as the method used for the qualitative analysis. Finally, we discuss the results and identify some general trends based on our area of interest (i.e. the social good).

### Related works

**Fig. 1:**
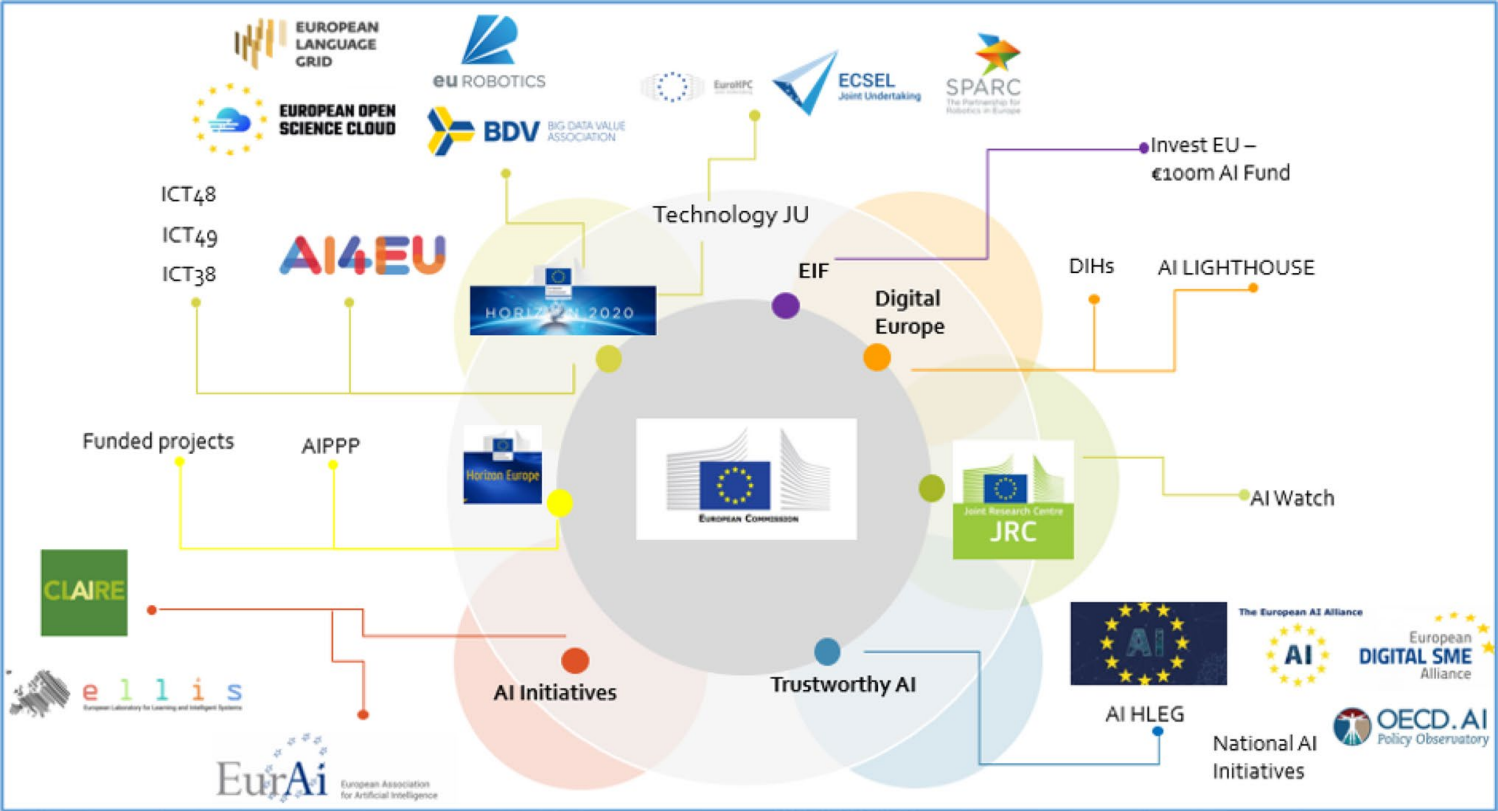
Representation of the initiatives promoted by the European Commission on AI. The figure is adapted from https://www.ai4europe.eu/Network-of-Excellence

This work connects to a large literature dealing with a variety of guidelines and frameworks, which have rapidly sprung up worldwide to promote the responsible and sustainable development of AI. Several studies carried out a comparative analysis to identify similarities and divergences among these initiatives. Examples include studies mapping keywords of different guidelines (Zeng et al. 2018) and broad scope reviews (Bradley et al. 2020; Jobin et al. 2019; Hagendorff 2020). Most of these studies consider a heterogeneous set of documents released by a variety of entities, including private companies, non-profit organisations and public institutions. Also, their main purpose is to study common ethical topics and their coverage across principles and guidelines issued in the last few years. Compared to these works, our study presents some points of contact, but also important distinctions. On the one hand, it shares the attention towards the ethical development of AI. But, on the other hand, it focuses on a more homogeneous set of documents (i.e. national strategies) which are all part of a challenging European strategy. So, rather than (dis)agreements on AI ethical principles, our focus is more on how these principles translate into plans and measures taken by the European countries. In particular, we do agree with Fjeld et al. (2020) that principles are better understood in their cultural, linguistic, geographic, and organisational context. Hence, investigating Europe’s AI strategy from the perspective of different Member States adds value to the study of the European AI landscape (Fig. 1).

### Materials and methods

To conduct our analysis of the investment plans stated in European national strategies, we selected the set of documents to be considered. We generated this selection based on the EU national strategies available from May 2020 to August 2020. According to AI Watch (Van Roy 2020) 22 nations were at least at the final draft of their strategy, a count that achieved 27 in the last revised report (Van Roy 2021). However, we have reduced the selection to 15 nations based on the following requirements:

1. The national strategy needs to be official. Neither draft nor action plans were considered.
2. Only AI strategies from Member States of the EU were included to ensure a common commitment towards the objectives of the Commission.
3. The documents need to be available in English to avoid language misinterpretations.

We gathered the national strategies selected in a list of documents alphabetically ordered in Table 2.

**Table 2:** List of the selected national strategies with a number of pages (PP)

Country	Year	Title	PP
Austria	2018	*ATM AT 2030: Artificial Intelligence Mission Austria 2030* (FMTI 2018)	16
Belgium	2019	*AI4Belgium (*AI4BELGIUM Coalition, 2019)	29
Czech republic	2019	*National Artificial Intelligence Strategy of the Czech Republic* (MITCR, 2019)	54
Denmark	2019	*National Strategy for Artificial Intelligence* (MIB, 2019)	74
Finland	2017	*Finland’s Age of Artificial Intelligence* (MEAE 2017)	76
France	2018	*For a meaningful Artificial Intelligence* (Villani, 2018)	154
Germany	2018	*Artificial Intelligence Strategy* (FEELS 2018)	45
Lithuania	2019	Lithuanian Artificial Intelligence Strategy: A Vision for the Future (MEI 2019)	22
Luxemburg	2019	*Artificial Intelligence: A Strategic Vision for Luxembourg* (GGDL, 2019)	28
Malta	2019	*Malta the Ultimate AI launchpad: A strategy and Vision for Artificial Intelligence in Malta 2030* (PSFDI 2019)	57
Netherlands	2019	*Strategic Action Plan for Artificial Intelligence* (MEC 2019)	64
Portugal	2019	*AI Portugal 2030* (INCoDe.2030, 2019)	36
Slovakia	2019	*Action Plan for the Digital Transformation of Slovakia* (Government of Slovakia, 2019)	78
Spain	2019	*Spanish RDI Strategy in Artificial Intelligence* (IMSU, 2019)	48
Sweden	2018	*National Approach to Artificial Intelligence*, (MEIn 2019)	12

**Fig. 2:**
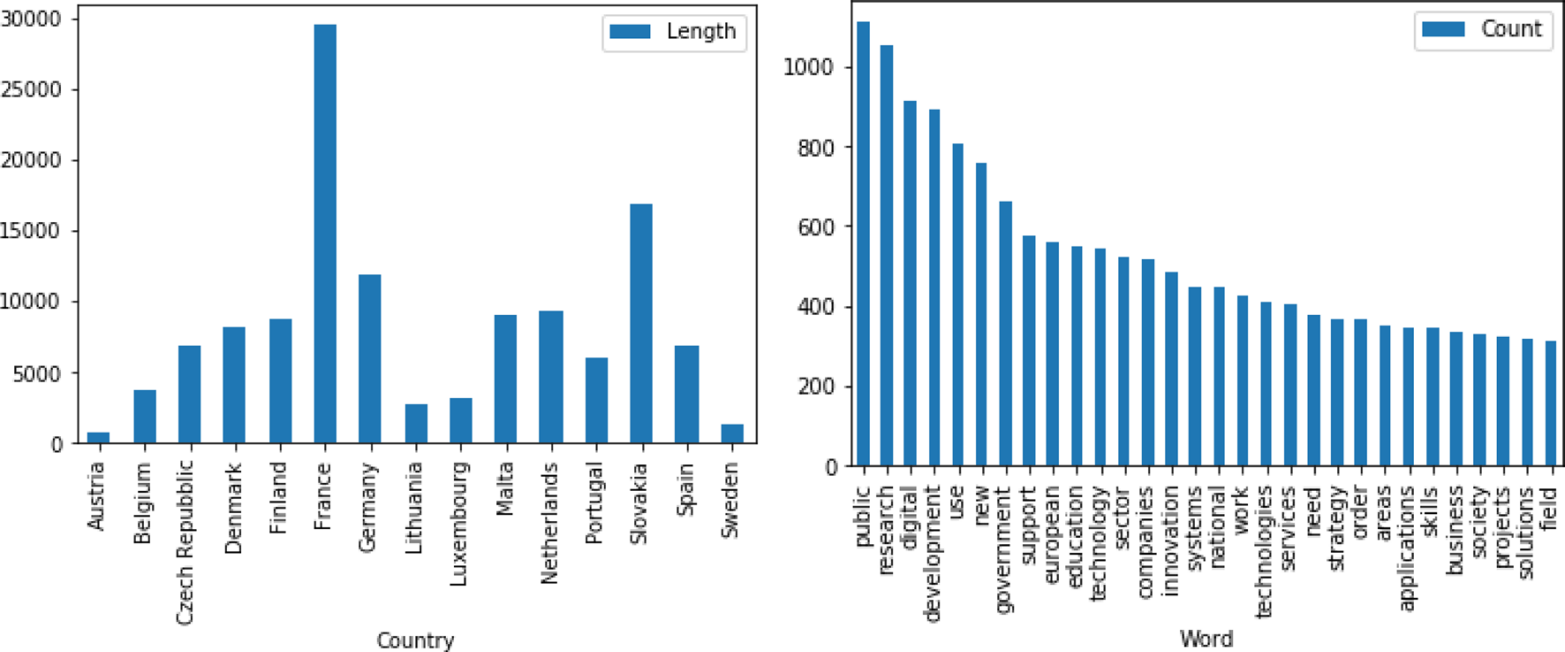
Lengths in unique tokens of National Strategies (**a**) and most frequent terms (**b**)

In the initial phase of our analysis, we extracted some quantitative information about our set of documents and the vocabulary employed. With this step, we aim to add the first layer of information describing the documents at large by means of simple statistics, such as term frequencies and terms’ relevance. We applied basic pre-processing techniques to remove useless terms (such as articles and prepositions) and broke up the text into essential units (tokens). This process highlighted the heterogeneity of our documents, whose length varies significantly ranging from 1000 to 30,000 tokens (for instance, see Austria vs France in Fig. 2a). In addition, we created a dictionary including a set of unique words^11^ to provide an overview of the vocabulary characterising the national strategies. Among the 10,262 unique terms, the 30 most frequent words in the whole corpus are reported in Fig. 2b. These include several terms that connect to the web of meanings characterizing the idea of socially good AI introduced in Sect. 1. Some of these relate to the public sphere (‘public’, ‘government’, ‘services’, ‘society’, ‘national’, ‘european’) while others are associated with a technological transition (‘research’, ‘development’, ‘innovation’, ‘education’, ‘work’, ‘companies’, ‘skills’). However, we observed that among the most frequent terms there are some general words lacking relevance for our research focus (e.g. ‘use’, ‘order’, ‘new’). Since this exercise was meant to give an overview of the vocabulary used by documents, we preferred to minimize our intervention, adding many constraints on the search and hence influencing the final result. An in-depth analysis of the context is hence required .

The core of our analysis consists in the application of grounded theory (Charmaz 2014) to explore our research question. In our case, this inductive approach is used to identify and collect meaningful pieces of text (i.e. the data) in the documents reporting specific budgets for investments. These data will be tagged with descriptive labels, hereafter referred to as codes, that summarise the concept or idea behind the text according to the open code method (Strauss and Corbin 1998). As compared to other qualitative approaches, grounded theory offers the flexibility to revisit the codes created by the researchers based on the emergence of new themes during the analysis of the text. The collection of data (i.e. portions of text) and codes (i.e. the label assigned) then formed our dataset.

**Fig. 3:**
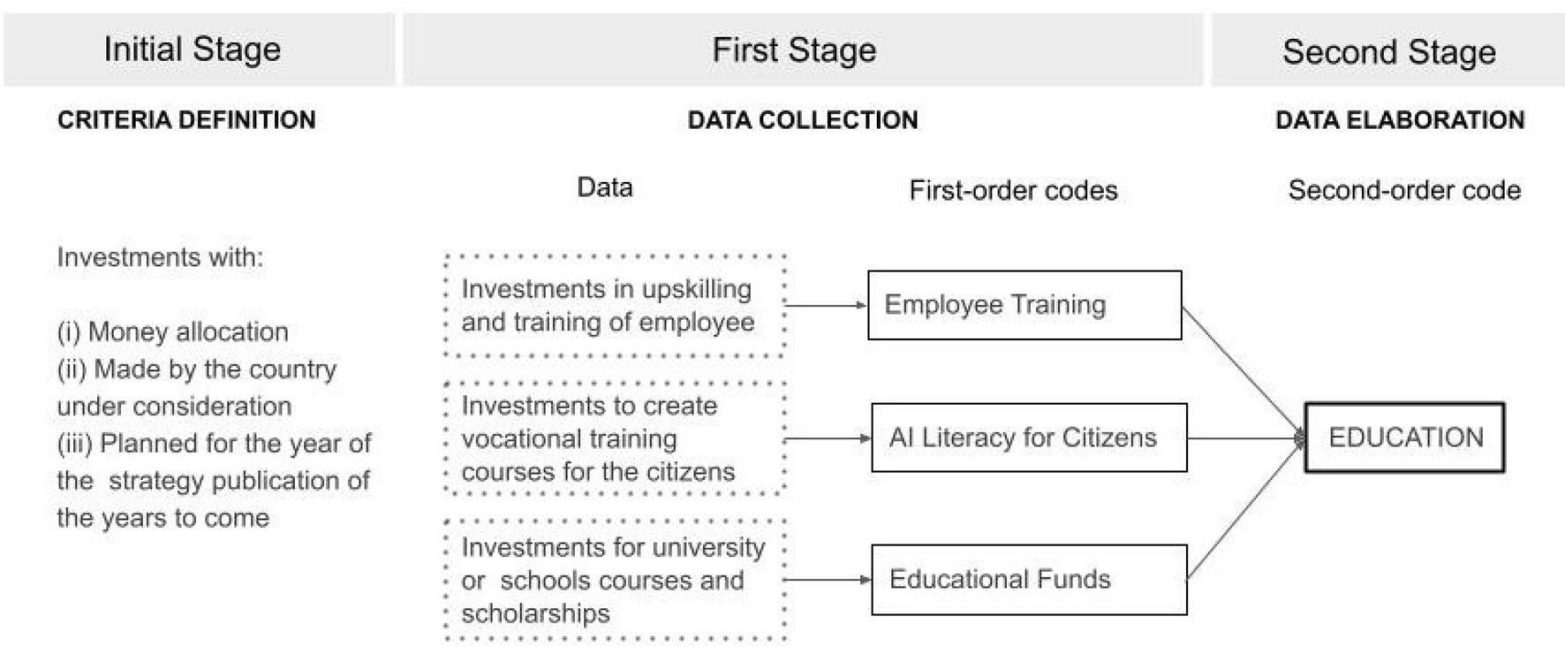
A visual representation of our two-stages qualitative analysis process and the creation of first and second-order codes with respect to the theme of education

We organized our analysis on a two-stage process involving two researchers. In the first stage, each researcher analysed independently the list of documents (see Table 2) to generate their own dataset with their own list of codes. In the second stage, the datasets were merged and the codes revised to obtain the final results. Figure 3 presents a sketchy representation of our process with respect to the theme of education.

To facilitate the analysis, before starting the process the researchers agreed on some common criteria in order to identify pieces of text of interest. The inclusion criteria were the following: (i) investments must include clear estimations in terms of money allocation; (ii) investments must be made by the country under consideration; and (iii) investments must be planned for the year of the publication or the years to come.

In the first stage, each researcher selected relevant portions of text based on the aforementioned criteria and assigned them to a code creating her own set of codes. During this process, the researchers were free to reiterate the process, adjust or create new categories of codes. This step was conducted individually to avoid that researchers were influenced by each other while interpreting the documents. Once completed the two datasets, the researchers cross-checked the results obtained to ensure a uniform joint dataset. Codes which were specific to only one of the two dataset were collaboratively revised and accepted/rejected according to the initial criteria. At the end of the control phase, five codes were deleted due to a lack of compatibility with the defined criteria (e.g. fundings returned entirely from the EU or investments made before the year of publication of the strategy).

**Fig. 4:**
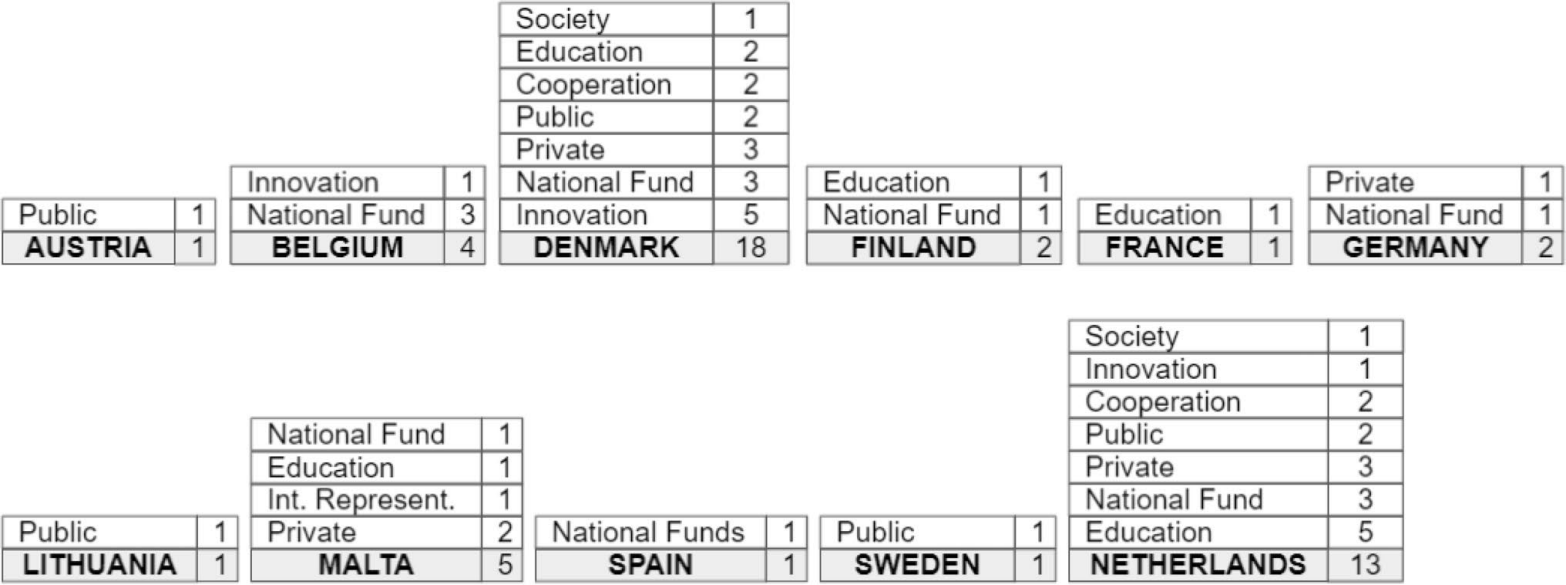
List of the 2nd order codes presented by European Member States in their AI national strategy with corresponding occurrences

In the second stage, the researchers created a new version of the dataset inspecting all the code to unify similar categories. The final version of the dataset is composed of 18 codes that cluster our 49 data related to investments. These codes referred also as *first-order codes*, describe directly the thematic content identified in the documents. We then organized the first-order codes into 8 higher-level categories by applying the axial coding methodology (Strauss and Corbin 1998). This second process generated the *second-order codes*, helping the researchers to generate new high-level relations between the codes. Table 3 represents the first and second-order codes and their descriptions while Fig. 4 presents the occurrences of second-order codes based on the Member States they refer to. The table includes the results from the analysis of 11 national strategies that have reported information related to investments based on our requirements. The complete version of the analysis, including the text selected and document details can be found in the appendix.

**Table 3:** Qualitative analysis of the investment area in the European AI national strategies

2nd ORDER CODES 1st order codes	*N* (#occ)	Description	NATIONS (#of occurrences)
SOCIETY	2		
Social Impact	1	Investment to evaluate the impact of AI on society and employment	Netherlands
Digital Welfare Solution	1	Investment to accelerate the dissemination of digital welfare solutions	Denmark
COOPERATION	3		
Public Collaboration	1	Funding to foster collaboration between public entities on AI research	Netherlands
Public–Private Collaboration	1	Funding to develop joint projects and collaborations between public and private sectors	Denmark
International Collaboration	1	Investment to foster international collaboration among AI researchers	Denmark
NATIONAL FUND	11		
Current Investment	6	Description of ongoing packages of investments in AI for general purposes	Belgium, Denmark (2) Germany, Malta, Spain
Future Investment	5	Projections of future money allocations or packages of investments that will conclude in the future	Belgium (2), Denmark, Finland, Netherlands
INNOVATION	7		
Digital Technology	4	Investments to research and develop new technologies and services to achieve a digital transformation	Belgium, Denmark (3)
Cybersecurity	1	Funding to improve the national cybersecurity	Denmark
Data Collection	1	Investment intended to improve the data quality and the public accessibility to the data set	Denmark
Supercomputing	1	Funding to acquire a new supercomputer to support the research	Netherlands
INTERNATIONAL REPRESENTATION	1		
National promotion	1	Investment to promote the visibility of the country as an opportunity for companies and research in AI	Malta
EDUCATION	12		
Employee Training	6	Investments for training, upskill, or facilitating access to new workers in the AI sector	Malta, Netherlands (5)
AI Literacy for Citizens	2	Fundings to support the general public (e.g. NEETs) in developing their digital competencies through the creation of vocational training	Denmark, France
Educational Fund	4	Investments to support and strengthen education in AI with the creation of scholarships, university programs, and higher education	Denmark, Finland, Netherlands (2)
PRIVATE	8		
Companies Investment	8	Investments made to launch, support, or co-support enterprise and start-ups	Denmark (3), Germany, Malta (2), Netherlands (2)
PUBLIC	5		
Investment for Local Administrations	1	Investment for local administrations (e.g. municipality and region) to improve their services	Denmark
AI Research	4	Investment in educational and research resources	Austria, Lithuania, Denmark, Sweden

### Limitations

Our analysis is bounded by some limits regarding the framing of our research questions and the corpus of the documents selected. For example, even if the EC provided some guidance (European Commission 2018a) in the definition of priorities and policy measures, each national strategy presents differences in the structure and details provided. Since there were documents which did not provide specific and exhaustive information on investments, it was not possible to run a full comparison. For this reason, we decided to consider only current or future investments with a specified budget. This choice was motivated by a twofold reason: on the one hand, to define in a clear way the area of interest for coding, on the other hand, to focus on concrete actions taken by the states, not merely general intentions. We believe that the presence of a budget represents a stronger commitment that the State takes towards the EU directives. However, we are aware that the findings of this research might not correctly represent the current status of the investment in the EU. Thus, future studies in this area may complement our findings with estimation from the market to have a more representative vision of the AI landscape in the EU.

## Results

According to the results, 11 national strategies have reported investment plans that meet our requirements. These include Austria, Belgium, Denmark, Finland, France, Germany, Lithuania, Malta, Spain, Sweden and the Netherlands. In the following subsections, we highlight the main findings distinguishing between general investment plans and investments with an explicit commitment to society (e.g. welfare solutions, education and social impact).

The table presents the 1st and 2nd order codes, their occurrences (#occ), and the countries reporting the code in their strategies with the related occurrences and a brief description (e.g. Employee Training 1 investment in Malta and 5 investments in the Netherlands).

Most of the national strategies (7 out of 11) report packages of investments in AI initiatives (National Fund). These investments vary depending on whether they refer to ongoing efforts (Current Investment) or future plans (Future investment). Their description is usually generic and reports total volumes, which often cover different areas of application (e.g. such as healthcare and life sciences). Some strategies provide figures which connect to the digital transformation (Innovation). For example, the Netherlands is investing EUR 18 million in a new national supercomputer (Supercomputing), while Denmark allocated DKK 1.5 billion (EUR 200 million) on cyber and information security (Cybersecurity) and DKK 250 million (EUR 33.5 million) in data quality and cross-sectoral cooperation on health data (Data Collection).

Another emerging trend regards investments in the private sector (Private), putting special attention to support start-ups and SMEs in the uptake of AI, as they make 99% of business in Europe (European Commission 2018b). Thus, it is clear that the early adoption of new technologies will help boost innovation and competition in the AI landscape. In some strategies, there are figures that refer more specifically to the public sector (Public). For instance, Denmark allocates resources for testing and deploying digital welfare solutions in municipalities and regions (Investment for Local Administration), while Austria, Lithuania, Denmark, and Sweden report investments in academic research (AI Research). Another interesting case regards Malta, which plans to spend EUR 1 million per annum to promote their international visibility and become an emerging hub for technologies in Europe (International Representation). Our findings on investments regarding the private and public sector well align with the estimates of investments made in 2018 in the EU (Nepelski and Sobolewski 2020), which suggested, among others, higher pro-capital investments for northern countries (e.g. Denmark, Ireland, Finland, Sweden and the Netherlands). Further evidence comes from a recent EC’s survey which found that 42% of European companies are already using AI (European Commission 2020b).

A few documents report quantified investments regarding actions related to society. For example, the Netherlands assigns funding to study the impact of AI on work and employment (Social Impact). Denmark specifies allocated resources for digital welfare solutions (DigitalWelfare Solution), which connect to a wider reform in the Public Sector aimed at contributing to better and more cohesive welfare services.

Regarding education, seven strategies propose economic plans. For example, Denmark and France reported investments to support the population in developing digital skills on AI and facilitate their integration into the new labour market. (AI Literacy for Citizens). The Netherlands describe multiple economic initiatives (5 codes) for training workers and promoting a learning culture in SMEs (Employee Training). As a reference, during 2018 just a few EU countries (e.g. Bulgaria 23.47% Luxembourg 2.9%, Slovenia 2.6% Croatia 2.23%) invested in corporate training with marginal movements in their national budget on AI (Nepelski and Sobolewski 2020). Moreover, along with Denmark and Finland, they propose concrete investments in higher education. For example, the Danish government set aside a pool of DKK 190 million (EUR 25.5 million) to cover all technical fields, including new technologies like AI (Educational Fund). Another interesting proposal regards the investments for cooperation. Indeed, the Netherlands reported an open call, worth EUR 2.3 million, that involved different public organisations to coordinate efforts in research on explainable, socially aware and responsible AI (Public collaboration).

## A discussion of social good in national strategies

In this section, we consider the collected results in the light of the policy directions put forward by the EC to understand to what extent National Strategies care about AI for social good. In particular, we focus on the macro-areas identified in Sect. 2.2 to classify the measures outlined in EC’s Policy documents for the achievement of the European AI strategy goals.^12^

*Beneficial innovation*

With respect to a beneficial use of AI, we did not find explicit figures claiming the use of AI to tackle social problems. While the Netherlands refers to specific investments for a social purpose, that is for studying the impact of AI on work and employment, other countries claim figures to improve the public sector. In particular, Denmark plans to disseminate digital welfare solutions at the national and local levels, while other countries target resources for the growth of AI research and development (Denmark, Austria, Sweden, and Lithuania).

Planning AI-related investments to improve the efficiency of public service can play an important role in promoting AI for Social Good. These for example can allow the state anticipate and target interventions in social and health care. However, similar actions need to consider potential harms that may derive from the application of AI in the public sector. For instance, some studies showed that healthcare risk-prediction algorithms can increase inequalities within society (Obermayer et al. 2019) and similar mechanisms can extend to a vast array of services to regulate a community of people or even a country (Cristianini and Scantamburlo 2020). To avoid potential risks for society (such as discrimmiation and intrusive surveillance), the introduction of AI in the public sector would need prevention mechanisms and measures to ensure that the risk is carefully considered and monitored. An example of such measures includes participatory design approaches which strive for citizens’ participation, in particular those who are part of marginalised groups, in the whole lifecycle of an AI system (e.g. for testing and collecting feedback). Even without reporting specific funds, some national strategies aim to involve citizens in the process of defining future applications of AI, especially those that will be deployed and used by public administrations (e.g. Austria plans to support societal discussion and increase the acceptance of AI, and Czech Republic aims to involve employees in technological transformation).

*Education and life-long learning*

Education and life-long learning identifies a cluster of measures aimed at preparing society for the transformation brought about by AI in the labour market. In our research, even if few national strategies reported investments in this domain (5 nations out of 11), our analysis shows high diversification investment in this area. Note that already in 2018 there was an estimation of 58% of the European budget in AI covering education-related areas (Nepelski and Sobolewski 2020). These plans mostly regard the re-training and upskilling of the population, and will play an important role in promoting a more inclusive and sustainable innovation. In particular, AI literacy and education can contribute to filling the gap created by the rapid growth in AI between the “producers”, who know the strengths and limits of this technology, and the “consumers”, who may lack knowledge about AI and be more exposed to harmful applications. This will lead, on one side, to new opportunities for citizens to develop AI-based competences at work and contribute to the digital transformation that will shape our society. On the other hand, a widespread knowledge about AI can lead to a faster acceptance of new technology and penetration in society, bringing to life the aim to improve the society that Europe is wishing for.

*Governance mechanisms*

An area of intervention which explicitly addresses the good of society is the introduction of governance mechanisms through the creation and adoption of ethics guidelines and legal frameworks. With respect to this policy area, we did not observe quantified investments. In particular, five national strategies (Belgium, Denmark, Luxembourg, Malta, Spain) state that they want to create an ethical committee to supervise the use and development of AI systems. Malta puts forward the proposal of a national AI certification program based on its Ethical AI Framework. However, all these propositions lack details about allocated resources. While some of these proposals build upon existing initiatives and investments schemes we expect to see further measures like the ambitious goal of Trustworthy AI cannot be achieved without costs. The setup of an appropriate ethical and legal framework is, in fact, a demanding effort which implies a long-term view and the mobilisation of huge resources (e.g. experts in different fields, new business processes, holistic assessment methodologies, audits, etc.). However, as of today, national strategies take a reflexive approach based on ethical principles and changes are to be made rather than defining clear rule-based systems (Radu 2021).

*Cooperation and dialogue among stakeholders*

Another set of policy recommendations aims to increase cooperation and dialogue among stakeholders to exchange best practices and facilitate the partnership. Creating a web of stable interactions among AI stakeholders can also optimise efforts targeting social good (e.g. projects facing global social or environmental challenges or sharing standards and good practices implementing ethical requirements). For example, the EC sets out the AI Alliance precisely to engage different stakeholders, including citizens, in a broad and open discussion of different aspects of AI. Another important example of international collaboration is the Global Partnership on AI (GPAI), funded in 2020 to undertake common AI projects and share mechanisms for multidisciplinary analysis and coordination.^13^

In our analysis we found only 2 national strategies allocating explicit resources for cooperation. The Netherlands, for example, reports investments to foster collaborations among public entities on explainable, socially-aware and responsible AI. The other case is Denmark, which specifies funds for both national and international collaborations. Building synergies between different countries or even different continents has important implications in the creation of a common ground for Trustworthy AI. Indeed, international collaborations can help overcome cultural barriers and build mutual understanding of how to ensure safe and ethical AI innovation at a global level (ÓhÉigeartaigh et al. 2020).

*Pan-European data and technological infrastructure*

In the effort of achieving the expected transformation for AI, the infrastructure and the computational capabilities play a fundamental role in making this plan possible. Actions such as ensuring the data quality, making datasets publicly available, and putting in place mechanisms to protect their security are necessary to maintain trustworthiness at all levels of the AI pipeline. While this lays down the ground to implement projects and initiatives for AI for social good, we identified only two nations involved in this process: Denmark and the Netherlands. The former reports the intention to publicly share and improve the quality of the data collected for weather and forecasts. They also commit to prevent cyber attacks by investing in strengthening their cybersecurity. The latter instead focuses on the acquisition of a new supercomputer to ensure the computing capacity necessary for their research. The lack of infrastructure consideration presented in the national strategies raises concerns over the realistic possibility of the implementation of these plans. Moreover, specific measures for cybersecurity and detecting biases are expected to preserve the EU principle of prevention of harm.

## Concluding remarks

In this paper, we explored the field of AI for Social Good from the perspective of European policies and, in particular, through the lens of EU national strategies. Starting from the goals and the policy recommendations put forward by the EC, we define three possible and complementary approaches to AI for Social Good: “as applications”, “as ethical principles” and “as policies”. In this paper, we focus on the latter approach, “AI for social good as policies”, to analyse which of the investments extracted from the national strategies could promote AI for Social Good in concrete ways. We discovered some general patterns of investments and more specific plans for the social good, such as those concerning education, vocational training, welfare services, and research cooperation on responsible AI. A general observation regards the imbalance between the vocabulary used by national strategies and the statements concerning money allocations. While terms associated with social good abound throughout the documents—e.g. ‘public’, ‘society’, ‘education’ and ‘services’ are among 30 most frequent terms in all documents—, money allocations in areas that can be strategic for the development of AI for social good are limited. This points to a gap between words and actions marking the general debate on AI ethics. On the one hand, declarations by states and private organizations seem to put responsible and beneficial AI on the top of their agenda, but, on the other hand, the actions needed to implement such priorities are not yet clearly formulated. In this regard, our analysis confirms that resource and money allocation is a good proxy for understanding to what extent an organization pushes ahead with AI for social good, going beyond slogans and empty rhetoric. In that sense, a natural way to progress the work presented in this paper would be a follow-up investigation on the status of planned investments and future allocation schemes.

Among policy directions suggested by the EC (see Sect. 2.2), only education attracted significant investments. However, the investments reported in this area are mostly concerned with the introduction of AI in high schools and workforce training. While these measures are fundamental for guaranteeing an equitable access to the opportunities offered by AI, further funding could extend to other EC’s policy recommendations such as the integration of ethics and humanities into AI and STEM curricula, the strengthening of the multidisciplinary research environment and the improvement of gender balance in computer science and engineering disciplines. These findings are aligned with those presented in Schiff (2021), who also provides recommendations to boost ethics and policy-oriented AI in education research and create a real impact for the public good.

Other categories of policy recommendations, such as cooperation and dialogue with stakeholders remain relatively new and few nations report concrete funding in this field. Close collaboration with stakeholders and representatives of vulnerable groups is of crucial importance in the development and deployment of AI, in particular when its application regards the public sectors and services that should be available for the whole society. Collaboration can also mean more opportunities for public–private partnerships that address societal and environmental challenges, for example, by funding hackathons and competitions.

With respect to governance mechanisms, we observed several good initiatives but no specific information about numeric investments. Considering the recent EC’s proposal for an AI regulation, we expect to see more concrete measures in this domain. In particular, we believe that more resources would be needed to develop testing facilities and sandboxes to allow AI researchers and practitioners assess compliance with AI regulation. However, we also warn that actions guided only by the goal of legal compliance may not be enough to internalise the intrinsic motivations which underpin the development of AI for social good and substantiate the application of ethical principles (see the second meaning of AI4SG in Sect. 2.1). Self-interests from companies and the market may sometimes find a way to take over the existing law or use ethics to delay the work on regulations (Nemitz 2018). For this reason investments in AI for social good should, on the one hand, be well regulated to avoid grey areas and overtaking interests from the market and, on the other, be more connected to produce long-lasting effects. In this regard, defining numerical expectation in the investments for social good could not predict the effectiveness of the measures taken. The efforts should perhaps be more directed upon common goals based on genuine European values. More solidarity among EU states, for example by financially supporting the sharing and reuse of knowledge and tools, would help more equitable and common progress, as well as the consolidation of the goals achieved. Indeed, the European view of a suitable and responsible AI should not imply that some countries move faster and further than others. Europe should have a unified approach towards AI for social good with the same active participation by all the parts involved. Note that the revised version of the EU coordinated plan stresses this point by suggesting, for example, sharing, developing and implementing actions on the national/regional level that proved to be successful in the other Member States (European Commission 2021b).

Moreover, a proportionality principle can ensure that resources and efforts are fairly distributed across all sectors, not only in AI. The development of an ethical and suitable approach to AI would be more credible if we address other issues, such as climate change, health and migration with the same intensity and strength. The use of AI for social and environmental issues, like the recent Covid-19 crisis, should not reduce our efforts in other directions such as guaranteeing access to health treatments and vaccines to all. In conclusion, the use of an “ethics narrative” should not be used to cover marketing or political interest but to support real actions to achieve not only a “Trustworthy AI” but also a culture of trustworthiness. It is essential that all stakeholders engage in this process. In particular governments and policy-makers need to provide means to put into practice AI for Social Good, but also the Society (i.e. the citizens) needs to remain aware and actively demand changes in favour of social and environmental well-being.

## Appendix

**Table:** 

2nd order code (OCC)		1st order codes (OCC)		Text selected and Nation of reference		Page	References
Society	2	Social impact	1	NL	The Ministry of Social Affairs and Employment and the NWO are investing approximately €3 million in research to gain more insight into the impact of digital technologies (such as AI) on work and employment	p. 31	*Strategic Action Plan for Artificial Intelligence* (MEC 2019)
		Digital welfare solution	1	DK	The government has also proposed a new investment fund to expedite the dissemination of digital welfare solutions. Together with initiatives already launched, the investment fund will have a total investment budget of DKK 410 million (EUR 63.1 million) for 2018–2022	p. 20	*National Strategy for Artificial Intelligence* (MIB, 2019)
Cooperation	3	Public collaboration	1	NL	There will be thematic research calls for public-public collaboration, such as a recent call from the Netherlands Organisation for Scientific Research (NWO), initiated by the Ministry of the Interior and Kingdom Relations and worth €2.3 million, on explainable, socially aware and responsible AI (closing date 5 November 2019)	p. 44	*Strategic Action Plan for Artificial Intelligence* (MEC 2019)
		Public–Private collaboration	1	DK	In 2019, Digital Hub Denmark will also spend up to DKK 7 million (EUR 930,000) to develop public–private collaboration models to, among other things, improve the possibilities for life-science businesses to use, for example artificial intelligence in connection with research into health data	p. 58	*National Strategy for Artificial Intelligence* (MIB, 2019)
		International collaboration	1	DK	In collaboration with researchers from the US and Australia, researchers from Aalborg University will develop algorithms that can manage systems associated with great uncertainty. Independent Research Fund Denmark has granted DKK 5.8 million (EUR 800,000) to the project	p. 69	*National Strategy for Artificial Intelligence* (MIB, 2019)
National Funds	11	Current Investment	6	BE	While many European countries now have an AI-focused strategy, Belgium does not yet. Having said that, multiple initiatives are now underway. Flanders has launched its AI strategy, planning to spend an annual EUR 30 m on implementing AI in companies, top strategic research and flanking measures such as education, outreach and ethics	p. 5	*AI4Belgium (*AI4BELGIUM Coalition 2019)
				DK	In collaboration with the social partners and representatives from higher education institutions, the government has set up a vocational adult education and training working group which, among other things, will advise on the competence needs of the labour market in the light of technological and digital developments. As part of this National Strategy for Artificial Intelligence, the working group will examine whether there is a need to launch initiatives on the basis of the development of artificial intelligence, for example in the form of new education programmes, analyses and development projects. An annual amount of DKK 5 million (EUR 670,000) has been allocated for initiatives by the working group Under the Strategy for Denmark’s Digital Growth, the government has allocated DKK 110 million (EUR 15 million) for the Digital Hub Denmark initiative, which, among other things, is to market and strengthen Denmark’s position as an attractive growth environment for artificial intelligence	p. 48	*National Strategy for Artificial Intelligence* (MIB 2019)
						p. 58	
				DE	In the 2019 federal budget, the Federation has taken the first step, allocating a total of €500 million to beef up the AI strategy for 2019 and the following years. Up to and including 2025, the Federation intends to provide around €3 billion for the implementation of the Strategy. The leverage effect this will have on business, science and the Länder will mean that the overall amount available is at least doubled	p. 6	*Artificial Intelligence Strategy* (FEELS 2018)
				MT	Financial support will be provided for AI-related research commercialisation programmes and technology development (including financial support for research, development and innovation) in Malta’s areas of Smart Specialisation (ICT as an Enabler, ICT Based Innovation, Tourism Product Development, Aviation and Aerospace, Health, Resource Efficient Buildings, High Value-Added Manufacturing, Aquaculture) through the Malta Council for Science and Technology (MCST) R&I FUSION Programme until 2020. The annual budget currently allocated to the R&I FUSION Programme is €2.2 m	p. 21	*Malta the Ultimate AI launchpad: A strategy and Vision for Artificial Intelligence in Malta 2030* (PSFDI 2019)
				ES	The contributions made by the state bodies financing RDI, CDTI, AEI, ISCIII, MINECO and MINCOTUR, to the activities contributing to AI have involved 457 actions worth almost 114 million euros in financing (subsidies and credits)47. These actions have been granted through the tools offered by the four State Programmes developed in the State Plans and their Strategic Actions included in the EECTI 2013–2020 and the Natural Language Technologies Plan (2016–2018 calls). In the same period for the R&D Framework Programme H2020 there was Spanish participation in 116 actions related to AI that obtained a funding of € 79.30 million	p. 20	*Spanish RDI Strategy in Artificial Intelligence* (IMSU 2019)
		Future Investment	5	BE	Based on a yearly per capita investment, to match Finland, France and Germany, our minimum ambition level should be EUR 80 million per year. This corresponds to at least EUR 1 billion by 2030 A few principles to ensure a sustainable implementation: ensuring continued trust from the public, a European approach, collaboration between all stakeholders, a grass-roots/community-led approach, focus on specific areas (such as healthcare/life sciences) and, lastly, daring to be ambitious and audacious. This will require an investment of at least EUR 1 billion by 2030	p. 28	*AI4Belgium (*AI4BELGIUM Coalition, 2019)
						p. 8	
				DK	With their declaration AI in the Nordic-Baltic region, the Nordic and Baltic countries have agreed to cooperate on artificial intelligence to secure digital skills, access to data based on common standards, ethical guidelines for artificial intelligence, and to promote the development of the technology in Europe. The strategy contains 24 initiatives. The government has earmarked DKK 60 million (EUR 9.2 million) for 2019–2027	p. 19	*National Strategy for Artificial Intelligence* (MIB 2019)
				FI	In the short term, innovation funding should be targeted to the following themes in particular, with €100 m as a permanent increase in innovation funding from 2019: − The application of artificial intelligence in different sectors and the development of business expertise − Enterprise-driven ecosystems and strategic projects, the funding models for which will make it possible for new actors to join flexibly	p. 53	*Finland’s Age of Artificial Intelligence* (MEAE 2017)
				NL	The Netherlands is already benefiting from European opportunities and will continue to focus on them. Within the European Horizon 2020 programme, €986 million was allocated to 580 AI-related projects from 2014 to 2017. Dutch parties received €61 million (6.1%) of this. Assuming the same share, a budget of €48 million per year will be available for Dutch parties in AI-related European projects in the coming years. This can only be achieved if Dutch parties are and remain well connected at the European level by highlighting and supporting European initiatives	p. 29	*Strategic Action Plan for Artificial Intelligence* (MEC 2019)
Innovation	7	Digital Technology	4	BE	Moreover, the Investment Pact, proposed by the Prime Minister, identified close to EUR 30 billion worth of investments in digital transformation	p. 5	*AI4Belgium (*AI4BELGIUM Coalition, 2019)
				DK	In the 2019 State Budget, the Danish government has allocated DKK 215 million (EUR 27 million) to Innovation Fund Denmark to conduct research into new technological possibilities. The Budget also allocates DKK 80 million (EUR 10.7 million) to the Independent Research Fund Denmark to conduct research into digital technologies, including artificial intelligence. As part of the funds allocated for research into new technological possibilities under Innovation Fund Denmark, DKK 100 million (EUR 13.4 million) will be earmarked for a national centre for research into new digital technologies This is a supplement to the DKK 295 million (EUR 45.4 million) allocated in the Finance Act 2019 from the research reserve for research into new technological possibilities and digital technologies and for a national centre for research into digital technologies More than DKK 600 million (EUR 80.5 million) have been set aside to develop technological services for Danish businesses for the period 2019–2020. This will supplement the funding that has been set aside under Innovation Fund Denmark, which also promotes new technological solutions, see focus area 3	p. 46	*National Strategy for Artificial Intelligence* (MIB 2019)
						p. 20	
						p. 53	
		Cybersecurity	1	DK	In the years to come, DKK 1.5 billion (EUR 200 million) will be invested in work by the Ministry of Defence on cyber and information security. Furthermore, six targeted strategies have been drawn up for work in the most critical sectors on cyber and information security in accordance with the principle of sector responsibility	p. 30	*National Strategy for Artificial Intelligence* (MIB 2019)
		Data Collection	1	DK	Up to 2023, a large number of datasets on weather observations and forecasts will be made freely available for everyone. This will make it possible for electricity plants, for example, to develop solutions that adjust the production of electricity to wind and weather conditions, or for businesses to develop new apps for consumers. With its Health Data Programme, the government has also earmarked DKK 250 mill. to ensure better data quality and databases and to strengthen cross-sectoral cooperation on health data	p. 33	*National Strategy for Artificial Intelligence* (MIB 2019)
		Supercomputing	1	NL	Ministerial departments have the opportunity to invest in AI-related research themselves. The Ministry of Health, Welfare and Sport has chosen to invest in Prevention and Big Data and this investment has been doubled by the National Science Agenda. To ensure that researchers have access to sufficient computing capacity, the Ministry of Education, Culture and Science is investing €18 million in a new national supercomputer at SURF	p. 27	*Strategic Action Plan for Artificial Intelligence* (MEC 2019)
International representation	1	National promotion	1	MT	The Maltese Government has placed the development of new emerging technological sectors such as AI as a national priority. Through Tech.mt, a public–private foundation tasked with promoting Malta as a leading global hub for emerging technologies, including AI, the Government will provide €1 m per annum in funding to raise the visibility of the country’s offering and promote Maltese AI businesses through various marketing activities, with participation in and sponsorship of leading global AI and tech summits in Malta and overseas	p. 21	*Malta the Ultimate AI launchpad: A strategy and Vision for Artificial Intelligence in Malta 2030* (PSFDI 2019)
Education	12	Employee Training	6	MT	The Investing in Skills programme, co-financed through the European Social Fund (ESF), will be operational until 30 June 2023 and has a budget of €5 m to help employed people develop and increase knowledge and skills through training	p. 37	*Malta the Ultimate AI launchpad: A strategy and Vision for Artificial Intelligence in Malta 2030* (PSFDI 2019)
				NL	A scheme that will structurally make more than €200 million available in the form of individual budgets for training and development will be presented to the House of Representatives after the summer of 2019. This ‘STAP’ (Labour Market Position Stimulus) budget replaces the current tax deduction scheme for training and is accessible to everyone up to the state pension age The Ministry of Social Affairs and Employment is developing a scheme to implement the Wiersma motion, which will structurally make €48 million a year available from 2020 to promote a learning culture in SMEs and the Heerma motion, which will make a total of €60 million available over a period of five years to provide additional support to the agricultural, hospitality and recreation sectors in order to attract more ‘BBL’ students (students in the ‘work-based’ VET learning pathway) With ‘MKB!dee’, the Ministry of Economic Affairs and Climate Policy is challenging SMEs to come up with ideas that will lead to more investment in the training and development of workers. While intended for all SMEs, the scheme focuses in particular on the challenges of technical SMEs and digitalisation. It relates to the broad effects of digitalisation, not to the ICT sector as such. In 2019, €7.5 million is available The Regioal MBO Investment Fund (Regionaal Investeringsfonds mbo) scheme provides €25 million a year until 2022 for projects that improve the connection of Senior secondary vocational education (MBO) to the labour market, for example, if the profession for which they offer training changes as a result of AI	p. 31	*Strategic Action Plan for Artificial Intelligence* (MEC 2019)
						p. 31	
						p. 31	
						p. 31	
						p. 32	
		AI Literacy for Citizens	2	DK	The tripartite agreement from October 2017 put focus on creating a vocational adult education and training system that is better geared to strengthening digital competences of the entire workforce and to adapting to the changing needs of the labour market. To support this, a transition fund of DKK 95 million (EUR 12.7 million) annually was set up. In addition to the transition fund DKK 8 million (EUR 1.1 million) is allocated to additional activities	p. 48	*National Strategy for Artificial Intelligence* (MIB 2019)
				FR	Under the Big Investment Plan 2018–2022, €15bn have been ring-fenced for vocational training, primarily for the benefit of low-skilled jobseekers and low-skilled young NEETs (not in employment, education or training). This budget forms the skills investment plan (PIC)	p. 91	*For a meaningful Artificial Intelligence* (Villani, 2018)
		Educational Fund	4	DK	The government has launched a talent programme to provide the most talented and motivated students with better opportunities and greater challenges, so they become even more skilled in their fields. The government will set aside a pool of DKK 190 million (EUR 25 million) to cover all technical fields, including new technologies like artificial intelligence	p. 45	*National Strategy for Artificial Intelligence* (MIB 2019)
				FI	The absence of applied studies is also evident in universities of applied sciences and in vocational education and training. However, as stated in the budget proposal, universities of applied sciences will be allocated five million euros for their RDI activities. This appropriation should be used in a targeted way in cooperation with business to create high added-value products and services, especially in the utilisation of artificial intelligence, robotics and digitalisation applications in various sectors	p. 50	*Finland’s Age of Artificial Intelligence* (MEAE 2017)
				NL	Commissioned by the Minister of Education, Culture and Science, the universities have drawn up sectoral plans for the STEM, Social Sciences and Humanities sectors. The Ministry will contribute an extra €70 million to this, and the periodic resources for profiling can also be used for this purpose The Ministry of Education, Culture and Science support the Education Innovation with ICT Acceleration Plan for all higher education institutions (universities of applied sciences and research universities) by making €15 million available over four years	p. 32	*Strategic Action Plan for Artificial Intelligence* (MEC 2019)
						p. 32	
Private	8	Companies Investments	8	DK	It is proposed to launch a pilot project in the form of an investment pool of DKK 20 million (EUR 3.1 million) over four years targeting companies with a business model based on artificial intelligence. The prerequisite for this is 50 percent financing from the private sector amounting to a total investment pool of DKK 40 million (EUR 6.2 million). The fund will be managed by the Danish Growth Fund The government will launch a pilot project in the form of an investment pool of DKK 20 million (EUR 2.7million) over four years, and this will be targeted at enterprises with a business model based on artificial intelligence. The fund will be managed by the Danish Growth Fund. The aim of the project is to build a bridge between investors and Danish businesses within artificial intelligence and thereby nurture the Danish market for artificial intelligence solutions Given the requirement for private co-financing, the initiative will have a leverage effect, as private capital is also invested in the businesses. If the private level of funding is assumed to be about 50%, about DKK 40 million (about EUR 5.4 million) will be invested in the development of Danish businesses based on artificial intelligence. The effect of investments will be regularly reviewed and the project will be adjusted accordingly	p 0.21	*National Strategy for Artificial Intelligence* (MIB 2019)
						p. 56	
						p. 57	
				DE	We are creating new funding opportunities for venture capital and venture debt and will launch a Tech Growth Fund Initiative. The Federal Government is continuing to use its successful, well-established funding instruments for start-ups and is also developing new instruments designed to strengthen the German venture capital and venture debt markets. This includes the new, independent KfW Capital equity entity, which is to increase the annual amount of investment KfW provides to the Venture Capital and Venture Debt Funds to €200 million by 2020, thereby making it easier for young, innovative and high-growth technology firms to secure financing for their start-up and growth stages	p. 24	*Artificial Intelligence Strategy* (FEELS 2018)
				MT	Malta Enterprise has launched various incentives schemes which can finance innovative AI undertakings with a viable business concept in their early stages of development. The incentive schemes will run through 2019 and 2020 and may be extended to the following years. Assistance includes: seed funding grants of up to €25,000 under Business START (B.Start). The total budget available for this scheme is currently €1 m per year Repayable advances structured as a mezzanine finance instrument to support start-ups with a proven business concept undertake initiatives linked to raising equity investment from third parties, procuring equipment and crowd-funding. Typical support is in the range of €200,000 and advances are repayable over a number of years	p. 23	*Malta the Ultimate AI launchpad: A strategy and Vision for Artificial Intelligence in Malta 2030* (PSFDI 2019)
						p. 23	
				NL	In order to stimulate technology-driven entrepreneurship, a new government-wide start-up and scale-up strategy was published in June 2018, with an implementation budget of €65 million The Limburg Business Development Fund of LIOF (the Regional Development Agency of the Province of Limburg) provided eight early-stage AI-related financings and grants totalling €3.8 million	p. 23	*Strategic Action Plan for Artificial Intelligence* (MEC 2019)
						p. 24	
Public	5	Investment for Local Administrations	1	DK	The initiatives will support the implementation of artificial intelligence in the public sector. The ambition of the government is to allocate to the municipalities and the regions DKK 200 million (almost EUR 27 million) to establish an investment fund to test and deploy new technologies and digital welfare solutions in municipalities and regions. Together with initiatives already launched, the fund will have a total investment budget of DKK 410 million (EUR 55 million) up to 2022	p. 53	*National Strategy for Artificial Intelligence* (MIB 2019)
		AI research	4	AT	Public funding at the federal level for AI research totalled to EUR 349.9 million between 2012 and 2017	p. 8	*ATM AT 2030: Artificial Intelligence Mission Austria 2030* (FMTI 2018)
				DK	Denmark is among the countries in the OECD with the highest public investment in research and development, measured in relation to GDP, and there are strong research environments within artificial intelligence. The total public research budget for 2019 is DKK 23 billion (EUR 3.1 billion)	p. 14	*National Strategy for Artificial Intelligence* (MIB 2019)
				LT	AI academic research projects also received EUR 6.5 million from the Ministry of Education and Science. Private investment came primarily from venture capital firms with varying origins including Lithuania, United States, France and Russia	p. 8	Lithuanian Artificial Intelligence Strategy: A Vision for the Future (MEI 2019)
				SE	In November 2017, the Knut and Alice Wallenberg Foundation (KAW) also announced that it would donate SEK 1 billion to AI research	p. 7	*National Approach to Artificial Intelligence*, (MEIn 2019)

## Footnotes

1 Conferences on this theme abounded in the past years and reached well-established communities in the AI field. See, for instance, the NeurIps 2018 workshop (https://aiforsocialgood.github.io/2018/) and ICLM 2019 AISG (https://aiforsocialgood.github.io/icml2019/index.htm).

2 As well as journal special issues, see also Cambridge University Press’ book series: https://www.cambridge.org/core/series/artificial-intelligence-for-social-good/4E54639036002106212C0A3812752C7C.

3 Consider, for example, multi-stakeholder forums like “AI4People” (https://www.eismd.eu/ai4people/) and “AI for Good” (https://ai4good.org/), United Nations’ platform “AI for Good Global Summit”, companies’ programme such as Google AI’s open call (https://ai.google/social-good/) and Microsoft’s AI for Humanitarian Action (https://www.microsoft.com/en-us/ai/ai-for-humanitarian-action) and Oxford Initiative AIxSDGs (https://www.sbs.ox.ac.uk/research/centres-and-initiatives/oxford-initiative-aisdgs).

4 See the OECD Observatory website: https://www.oecd.ai/countries-and-initiatives.

5 The AI Watch is an initiative of the European Commission (EC) jointly developed by the EC Joint Research Centre (JRC) and the Directorate General for Communications Networks, Content and Technology (DG CONNECT). See more at https://knowledge4policy.ec.europa.eu/ai-watch_en.Note that another relevant monitoring activity is conducted by the OECD (Organisation for Economic Cooperation and Development), an intergovernmental economic organisation aiming to encourage economic progress and world trade https://www.oecd.org/. In particular, the OECD AI Policy Observatory aims at collecting data on AI worldwide. See more at https://oecd.ai/.

6 The Digital SME Alliance is a network of ICT small and medium sized enterprises in Europe. See more at https://www.digitalsme.eu/.

7 Claire AI is a network of confederation of Laboratories for Artificial Intelligence research in Europe. See more at https://claire-ai.org/

8 Ellis (European Laboratory for Learning and Intelligent Systems) is an European network for research including programs for PhD. See more at https://ellis.eu/.

9 EurAI (the European Association for Artificial Intelligence or ECCAI) represents the European community in Artificial Intelligence. See more at https://eurai.org/.

10 The AI4EU is a European project funded by Horizon 2020 aiming to create a platform for collaboration between the different stakeholders in Europe. The platform is present at https://www.ai4eu.eu/ and includes the Observatory on Society and AI. See more at https://www.ai4eu.eu/observatory.

11 The dictionary was created by using the Gentism library available at the following link: https://radimrehurek.com/gensim/.

12 Note that, given the nature of the documents analysed, our discussion rests upon a policy-based approach to AI for Social Good (what we called “AI for Social Good as policies” in Sect. 2.1).

13 For more information about GPAI see the website of the initiative: https://gpai.ai
